# Bone Metastases Pattern in Newly Diagnosed Metastatic Nasopharyngeal Carcinoma: A Real-World Analysis in the SEER Database

**DOI:** 10.1155/2020/2098325

**Published:** 2020-07-16

**Authors:** Xiaojing Yang, Hanru Ren, Weiwei Yu, Hongling Li, Xinmiao Yang, Jie Fu

**Affiliations:** ^1^Department of Radiation Oncology, Shanghai Jiao Tong University Affiliated with Sixth People's Hospital, No. 600, Yishan Road, Shanghai 200233, China; ^2^Department of Orthopedics, Shanghai Pudong Hospital, Fudan University, Pudong Medical Center, Shanghai 201300, China

## Abstract

**Objective:**

To evaluate the prevalence rate and survival situation of bone metastases in initial nasopharyngeal carcinoma (NPC) patients and the hazard and forecast elements of bone metastases NPC patients. *Patients and Methods*. The data collected from Surveillance, Epidemiology, and End Results (SEER) program between 2010 and 2016 were evaluated. Univariate and multivariable logistic analysis and the Cox regression were carried out to estimate predictors and elements of the being of bone metastases at diagnosis, respectively. The overall survival of different subgroups were appraised by log-rank tests and the Kaplan–Meier analysis.

**Results:**

Factors including male sex, higher N stage, presence of liver, and brain or lung metastases were largely related to the occurrence of bone metastases. The median survival time for bone metastasis NPC patients was 14.0 months. A factor of more than one primary sequence number predicted worse survival.

**Conclusion:**

The data offer corresponding risks and prognostic indicators of bone metastases for NPC patients.

## 1. Introduction

Nasopharyngeal carcinoma (NPC) is a rare malignant tumor with a high geographic risk and a serious risk of distant metastasis. The annual incidence rate of NPC is between 0.15% and 0.5% in Southeast Asia [1]. Although early NPC can be cured, patients are usually in advanced stage at the time of initial diagnosis. It has been reported that about 15% of patients with NPC have distant metastases at the time of initial diagnosis [2]. The TNM staging is a well-accepted standard for predicting the prognosis of NPC. However, for the patients who have distant metastasis at the time of initial diagnosis, more accurate prognostic indicators are in highly demand.

Bone is one of the most common sites of distant metastases in NPC patients [3]. Bone metastases could bring about pathological fractures and pain, which reduce the life quality of patients [4]. It is reported that the prevalence of bone metastases in NPC is 54%-80% [5]. The survival time of patients with NPC who have distant metastases at initial diagnosis varies greatly [6, 7]. Early detection and treatment can prevent bone-related complications such as fractures and relieve the symptoms and prognosis of patients [5]. At present, there is no clear screening guide for testing bone metastases in NPC patients. Early identification of risk factors for bone metastases allows thorough examination of high-risk patients with bone metastases. These patients could obtain in-time treatment at an early stage. Several previous research displayed the prognostic indicators for NPC patients with bone metastases in China [3, 5, 8, 9]. They reported that NPC patients with bone metastases who are of higher age, of higher N stage, of high serum lactic dehydrogenase levels, with anemia, with multiple bone metastases sites, and without radiotherapy had worse survival. Due to the ethnic and geographical differences in NPC patients, it would be more evident to explore the relevant data in Western countries.

The Surveillance, Epidemiology, and End Results (SEER) program of the National Cancer Institute was established in 1973 and offers a significant data source for epidemiological analysis [10]. With the application of the SEER database, this research was designed to define the prevalence and risk elements of bone metastases in the initial diagnosis of NPC patients in areas out of Asian, such as the United States, and to define the risk factors and overall survival (OS) for these patients.

## 2. Material and Methods

### 2.1. Ethical Considerations and Data Availability Statement

The patient consent was not required to use the data in SEER database. Our research is in line with the Helsinki Declaration and related ethics. Demographic and tumor data can be obtained directly from the SEER database.

### 2.2. Cohort Definition

**Figure 1:**
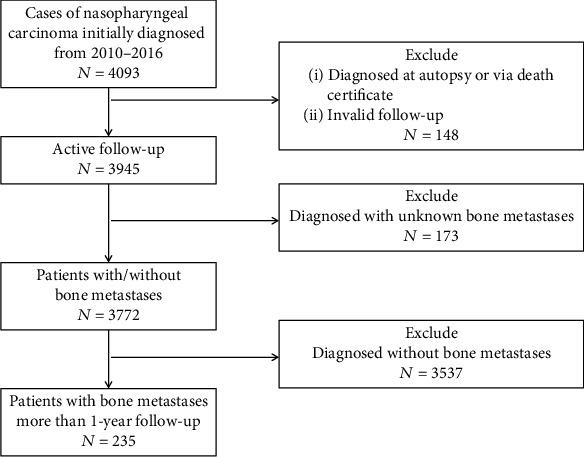
Flowchart of patient inclusion in this cohort study.

The patient data from 2010 to 2016 were acquired from the SEER database. The SEER∗ Stat software (Version 8.3.5, http://seer.cancer.gov/seerstat/download) was used to define inclusion criteria of NPC patients: (1) with a clear pathological diagnosis; (2) diagnosed from 2010 to 2016; (3) the stage of TNM of the patients follows the seventh edition of AJCC; and (4) the survival time is clear. Excluding cases with only autopsy or death certificates, invalid follow-up, and unknown bone metastases and NPC cases with bone metastases were eventually obtained. The detailed flow chart is shown in Figure 1.

### 2.3. Parameters

The demographic data included age (0-24, 25-49, 50-74, and ≥75 years), gender (male, female), race ((white, black, and others (American Indian/Alaska Native or Asian/Pacific Islander)), insurance status (insured, uninsured, or unknown), and marital status (married, single, or unknown). The clinical properties incorporated T stage (T0, T1, T2, T3, T4, and unknown) and N stage (N0, N1, N2, N3, and unknown), organ metastases including liver (none, yes, and unknown), lung (none, yes, and unknown), and brain (none, yes, and unknown), and sequence number (one primary only, others). And the data also included the survival status and time of each patient.

### 2.4. Statistical Analysis

Risk factors of newly diagnosed NPC patients with bone metastases were primarily determined by univariate logistic regression. If the results turned out statistically significant (*P* < 0.05), then the multivariate logistic regression was used for further analysis. The OS was defined as the time from the diagnosis to death, which is the main result of survival analysis. Differences in survival were dissected by the Kaplan–Meier analysis and log-rank test. A multivariate Cox proportional hazard regression was performed by analyzing the above factors. The statistical analysis was performed by SPSS 23.0 (IBM Corporation, Armonk, NY). *P* < 0.05 was known as statistically significant.

## 3. Results

### 3.1. Demographic and Clinical Characteristics

In the duration of 2010 and 2016, 3,772 NPC patients in the SEER database met our screening criteria (Figure 1). 4.1% of these people were younger than 25 years old. 22.1% of patients were between the ages of 25 and 49. A large percent referring 58.2% of the patients were between 50 and 74 years old, and 10.3% were older than 75 years. The ratio of women to men is about 1 : 2.4. For the ethnic information, 47.7% are white, and 12.1% are black. The remaining 40.2% are American Indian/AK Aboriginal and Asian/Pacific Islander. Most of them are married (56.2%) and insured (62.9%). As for the TNM stage, the T1 and N1 stages accounted for 27.5% and 27.0%, respectively. On the other hand, the patients presented liver, brain, or lung metastases accounting for 7%, 1.0%, and 4.2%, respectively. 83.7% of the patients had one major serial number. Demographic and clinical data details are displayed in Table 1.

### 3.2. Prevalence of Bone Metastases

In the entire cohort study, the prevalence of bone metastases with the initial diagnosis of NPC was 6.2% (235/3,772) (Figure 1). An average follow-up time was 14.9 months for all 235 patients with bone metastases.

### 3.3. Risk Factors for Spreading Bone Metastases

Univariate analysis showed that different factors were significantly associated with the prevalence of bone metastases. Patients that were male (OR = 1.765, 95% CI: 1.269-2.456, *P* ≤ 0.001), uninsured (OR = 1.481, 95% CI: 1.123-1.953, *P* = 0.005), and had liver, brain, and lung metastases (OR = 37.768, 95% CI: 25.457-55.991, *P* ≤ 0.001; OR = 18.708, 95% CI: 9.748-35.904, *P* ≤ 0.001; and OR = 11.958, 95% CI: 8.367-17.089, *P* ≤ 0.001), and not only one primary sequence number (OR = 1.849, 95% CI: 1.192-2.869, *P* = 0.006) were more likely to have bone metastases. Multivariate logistic regression analyses that showed male, presence of liver, and brain or lung metastases at initial diagnosis were positively related to bone metastases (Table 1).

### 3.4. Survival Analysis and Prognostic Factors for Bone Metastases

**Figure 2:**
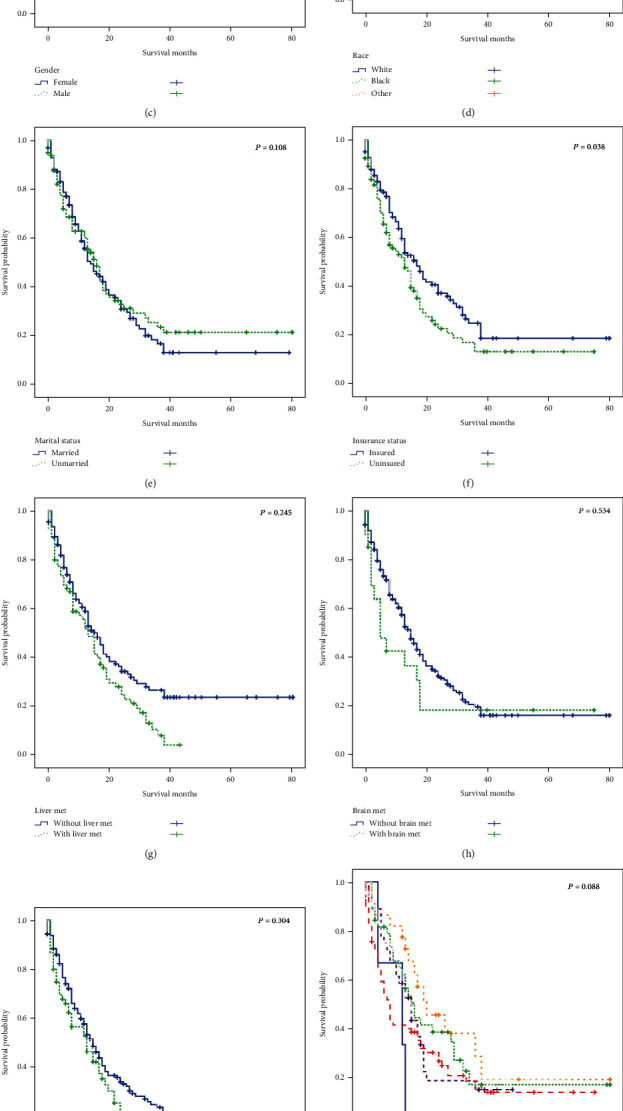
The Kaplan–Meier analysis of overall survival among patients diagnosed with nasopharyngeal carcinoma with initial bone metastases ((a), overall), stratified by age (b), gender (c), race (d), marital status (e), insurance status (f), liver metastases (g), brain metastases (h), lung metastases (i), T stage (j), N stage (k), and sequence number (l).

At the end of follow-up, 67.2% (*N* = 158) of NPC patients who had bone metastases at the time of initial diagnosis died. According to our univariate analysis model, the median OS for these NPC patients was 14.0 months (95% CI: 11.478-16.522 months, Figure 2(a)). Older age (Figure 2(b)), insurance status (Figure 2(f)), and more than one primary sequence number (Figure 2(l)) were negatively correlated with OS. On the other hand, gender (Figure 2(c)), race (Figure 2(d)), marital status (Figure 2(e)), liver, brain, and lung metastases (Figure 2(g)–2(i)), and TNM stage (Figure 2(j) and 2(k)) showed no significant relationship with prognosis.

By using the multivariate Cox regression, the patients only with primary NPC (HR = 1.868, 95% CI: 1.061-3.287, *P* = 0.030) had better OS than the other groups of which the median survival time was 15 months, while the other group was 7 months (Table 2).

## 4. Discussion

At the moment, this research is the largest scale of analysis on bone metastases in NPC in the United States. The SEER database was carried out to analyze the prevalence and survival rate of the newly diagnosed bone metastasis in NPC in the United States between 2010 and 2016.

According to the reports, compared with breast cancer, lung cancer, and prostate cancer, the prevalence of bone metastasis in patients with NPC is relatively low [10–12]. This study showed that 6.2% of NPC patients had bone metastases at the initial diagnosis, consistent with the study conducted by Yang et al. (7.7%) [13] while other studies reported the opposite results [14, 15]. This could have partly resulted from various detection methods used to detect the rate of bone metastases in NPC patients [14, 16]. However, the method used to identify bone metastases in these bone metastasis NPC patients in the SEER database was hard to define. Only a few studies on risk factors for bone metastasis in patients with NPC were reported in the United States. On the other hand, the related studies were reported in China, one of which showed that sex, C-reactive protein, neutrophils, platelets, hemoglobin, and other factors were notably related to the progress of bone metastases [13]. In addition, in this study, the number/location of lymph node metastases was also related to the development of bone metastases. These indicators could provide clinical value for NPC patients to predict a high risk of developing bone metastases. For these NPC patients in high risk, the bone should be further examined to check bone metastases.

In addition, identifying prognostic factors related to bone metastases in NPC can help the physicians with providing personalized treatment strategies for different patients. It also improves the quality of life and promotes a good prognosis for the patients. Our research demonstrated for the first time that the sequence number is related to OS in bone metastasis NPC patients. Among the NPC patients with bone metastases at the initial diagnosis, the prognosis of patients with only one mass at the primary diseased site was better than that of patients with multiple masses, as a result of the activity of tumor cells. Multiple masses at the primary site imply that the tumor cells are active and more prone to metastasize, which bring a poor prognosis for patients. Previous studies of patients with NPC in the SEER database showed that the race [17, 18], marital status [19], and age [20, 21] were associated with the prognosis of the patients. However, this is based on patients who have not had distant metastases. In addition, this study showed that the above factors have no significant relationship with patient prognosis. This could have resulted from multiple reasons. In the first place, NPC is not a common tumor in the United States as 70% of new NPC occur in East and Southeast Asia each year [22], and the number of cases collected in this study is not enough. As a result, the stratified analysis of the relationship between different races and prognosis in patients with bone metastases of NPC may bring bias to the study. Moreover, for patients who have had a distant metastasis, marital status may have little effect on their treatment and prognosis. This could be illustrated in that the OS of these advanced patients was short and no long-term relationship was observed. Besides, regarding to the age in this study, it was refined into four stages, and the results turned out that patients older than 75 years have a significantly worse prognosis. This is consistent with the research of Huang et al. [21], which showed that senior age is a risk factor for poor prognosis. Further investigations are required with a large number of patients admitted in the study.

There were also limitations in this research. This is a retrospective analysis that may bring bias to the results. Meanwhile, NPC has a low prevalence in the United States, and the sample size is not large enough. Besides, the detection methods for bone metastasis in these cases are not included so that the differences between multiple methods are required to be detected. In addition, it would be better to combine the data in the SEER database with the data of NPC patients in East and Southeast Asia, which would give more comprehensive information to analyze the global NPC patients.

## 5. Conclusion

To conclude, in newly diagnosed NPC patients, the prevalence of bone metastases is close to 6.2%. Bone metastases could reduce the survival rate of NPC patients. Especially at the time of initial diagnosis, further detection of the bone should be considered as a routine examination of male NPC patients. Our data identify a series of risk and prognostic factors for NPC patients with bone metastases and provide proof to realize early detection of bone metastases. This would be beneficial to the clinicians to choose the appropriate treatment with better patient survival.

## Figures and Tables

**Table 1:** Univariate and multivariable logistic regression for analyzing the demographic and related clinical characteristics for developing bone metastases in patients diagnosed with initial NPC (diagnosed 2010–2016).

Subject characteristics	No. of NPC patients		Univariable		Multivariable	
	Without bone met (*N*, %)	With bone met (*N*, %)	OR (95% CI)	*P*	OR (95% CI)	*P*
Age						
0-24	142 (92.8)	11 (7.2)	1 (reference)	1.0	1 (reference)	1.0
25-49	757 (90.8)	77 (9.2)	1.031 (0.535-1.988)	0.972	1.295 (0.528-2.881)	0.527
50-74	2066 (94.1)	129 (5.0)	0.800 (0.423-1.516)	0.494	0.978 (0.447-2.140)	0.956
75+	372 (95.4)	18 (4.6)	0.620 (0.286-1.346)	0.227	0.813 (0.314-2.103)	0.667
Gender						
Female	1063 (95.9)	46 (4.1)	1 (reference)	1.0	1 (reference)	1.0
Male	2474 (92.9)	189 (7.1)	1.765 (1.269-2.456)	0.001	1.676 (1.134-2.478)	0.010
Race						
White	1703 (94.7)	96 (5.3)	1 (reference)	1.0	1 (reference)	1.0
Black	425 (93.0)	32 (7.0)	1.336 (0.883-2.021)	0.171	0.893 (0.537-1.483)	0.662
Others	1409 (92.9)	107 (7.1)	1.347 (1.014-1.790)	0.040	1.003 (0.711-1.415)	0.985
Marital status						
Married	1996 (94.1)	125 (5.9)	1 (reference)	1.0	1 (reference)	1.0
Single	1321 (93.2)	96 (6.8)	1.160 (0.882-1.528)	0.298	0.946 (0.664-1.348)	0.759
Unknown	220 (94.0)	14 (6.0)	NA	NA	NA	NA
Insurance status						
Insured	2249 (94.7)	125 (5.3)	1 (reference)	1.0	1 (reference)	1.0
Uninsured	1142 (92.4)	94 (7.6)	1.481 (1.123-1.953)	0.005	1.256 (0.903-1.746)	0.176
Unknown	146 (90.1)	16 (9.9)	NA	NA	NA	NA
T stage						
T0	19 (86.4)	3 (13.6)	1 (reference)	1.0	1 (reference)	1.0
T1	1001 (96.3)	38 (3.7)	0.240 (0.068-0.848)	0.027	0.455 (0.096-2.149)	0.320
T2	525 (96.0)	22 (4.0)	0.265 (0.073-0.964)	0.044	0.457 (0.094-2.229)	0.333
T3	594 (94.3)	36 (5.7)	0.384 (0.109-1.358)	0.137	0.632 (0.133-3.018)	0.566
T4	715 (91.1)	70 (8.9)	0.620 (0.179-2.147)	0.451	0.917 (0.197-4.266)	0.911
Unknown	683 (91.2)	66 (8.8)	NA	NA	NA	NA
N stage						
N0	797 (96.6)	28 (3.4)	1 (reference)	1.0	1 (reference)	1.0
N1	970 (95.1)	50 (4.9)	1.467 (0.915-2.352)	0.111	1.086 (0.630-1.873)	0.767
N2	766 (93.1)	57 (6.9)	2.118 (1.333-3.366)	0.001	1.844 (1.085-3.136)	0.024
N3	405 (89.0)	50 (11.0)	3.514 (2.179-5.667)	<0.05	2.052 (1.166-3.611)	0.013
Unknown	599 (92.3)	50 (7.7)	NA	NA	NA	NA
Liver Met						
None	3488 (95.8)	152 (4.2)	1 (reference)	1.0	1 (reference)	1.0
Yes	48 (37.8)	79 (62.2)	37.768 (25.457-55.991)	<0.05	23.742 (15.261-36.938)	<0.05
Unknown	1 (20.0)	4 (80.0)	NA	NA	NA	NA
Brain Met						
None	3519 (94.4)	209 (5.6)	1 (reference)	1.0	1 (reference)	1.0
Yes	18 (47.4)	20 (52.6)	18.708 (9.748-35.904)	<0.05	10.372 (4.505-23.676)	<0.05
Unknown	0 (0.0)	6 (100.0)	NA	NA	NA	NA
Lung Met						
None	3431 (95.3)	171 (4.7)	1 (reference)	1.0	1 (reference)	1.0
Yes	99 (62.3)	59 (37.3)	11.958 (8.367-17.089)	<0.05	6.027 (3.835-9.473)	<0.05
Unknown	7 (58.3)	5 (41.7)	NA	NA	NA	NA
Sequence number						
One primary only	2946 (93.3)	212 (6.7)	1 (reference)	1.0	1 (reference)	1.0
Others	591 (96.3)	23 (3.7)	1.849 (1.192-2.869)	0.006	1.298 (0.777-2.167)	0.319

All factors with unknown data were removed from the Cox and Kaplan–Meier model. NPC: nasopharyngeal carcinoma; IQR: interquartile range; Met: metastases; NA: not available.

**Table 2:** Multivariable Cox regression for analyzing the prognosis factors for primary NPC with bone metastases.

Subject characteristics	No. of NPC patients with bone metastases		Survival, median (IQR), mo	HR (95% CI)	*P*
	Overall	Dead (*N*, %)			
Age					
0-24	11	6 (54.5)	24 (7.903-40.097)	1 (reference)	1.0
25-49	77	47 (61.0)	18 (14.907-21.093)	1.298 (0.501-3.361)	0.591
50-74	129	90 (69.8)	13 (9.617-16.383)	2.095 (0.805-5.448)	0.129
75+	18	15 (83.3)	7 (3.425-10.575)	3.896 (1.291-11.754)	0.016
Gender					
Female	46	30 (65.2)	19 (10.596-27.404)	1 (reference)	1.0
Male	189	128 (67.7)	13 (10.739-15.261)	1.386 (0.885-2.171)	0.153
Race					
White	96	64 (66.7)	13 (7.163-18.837)	1 (reference)	1.0
Black	32	22 (68.8)	13 (9.317-16.683)	1.260 (0.723-2.195)	0.415
Others	107	72 (67.3)	15 (11.383-18.617)	1.177 (0.805-1.723)	0.400
Marital status					
Married	125	85 (68.0)	14 (9.798-18.202)	1 (reference)	1.0
Unmarried	96	62 (64.6)	16 (12.550-19.450)	0.924 (0.644-1.326)	0.668
Unknown	14	11 (78.6)	NA	NA	NA
Insurance status					
Insured	125	78 (62.4)	17 (13.007-20.993)	1 (reference)	1.0
Uninsured	94	67 (71.3)	13 (9.003-16.997)	1.338 (0.934-1.915)	0.112
Unknown	16	13 (81.3)	NA	NA	NA
T stage					
T0	3	3 (100)	12 (0.000-24.803)	1 (reference)	1.0
T1	38	27 (71.1)	16 (11.842-20.158)	0.475 (0.130-1.727)	0.258
T2	22	14 (63.6)	20 (9.457-30.543)	0.329 (0.084-1.286)	0.110
T3	36	28 (77.8)	15 (10.623-19.377)	0.567 (0.154-2.085)	0.392
T4	69	55 (79.7)	8 (5.829-10.171)	0.699 (0.194-2.518)	0.584
Unknown	66	30 (45.5)	NA	NA	NA
N stage					
N0	28	22 (78.6)	12 (0.332-23.668)	1 (reference)	1.0
N1	50	39 (78.0)	13 (9.850-16.150)	1.093 (0.603-1.980)	0.769
N2	57	42 (73.7)	17 (13.379-20.621)	0.919 (0.543-1.555)	0.754
N3	50	39 (78.0)	13 (6.447-19.553)	1.208 (0.669-2.179)	0.531
Unknown	50	16 (32.0)	NA	NA	NA
Liver Met					
None	152	91 (59.9)	15 (12.02-17.980)	1 (reference)	1.0
Yes	79	64 (81.0)	13 (9.811-16.189)	1.375 (0.947-1.995)	0.094
Unknown	4	3 (75.0)	NA	NA	NA
Brain Met					
None	209	138 (66.0)	15 (12.429-17.571)	1 (reference)	1.0
Yes	20	15 (75.0)	5 (0.772-9.228)	1.399 (0.771-2.540)	0.270
Unknown	6	5 (83.3)	NA	NA	NA
Lung Met					
None	171	110 (64.3)	15 (12.048-17.952)	1 (reference)	1.0
Yes	59	45 (76.3)	13 (6.425-19.575)	1.361 (0.920-2.014)	0.123
Unknown	5	3 (60.0)	NA	NA	NA
Sequence number					
One primary only	212	138 (65.1)	15 (12.305-17.695)	1 (reference)	1.0
Others	23	20 (87.0)	7 (0.000-15.307)	1.868 (1.061-3.287)	0.030

All factors with unknown data were removed from the Cox and Kaplan–Meier model. NPC: nasopharyngeal carcinoma; IQR: interquartile range; mo: months; Met: metastases; Surg: surgical treatments; NA: not available.

## Data Availability

The data used to support the findings of this study are available from the corresponding author upon request.
